# Supplementary material for: A Binary Prototype for Time-Series Surveillance and Intervention
Source: medRxiv. 2025 Feb 5:2025.02.03.25321613. Preprint. [Version 1] doi: 10.1101/2025.02.03.25321613 (PMC11838624; doi:10.1101/2025.02.03.25321613)
Supplement: Supplement 1 [file NIHPP2025.02.03.25321613v1-supplement-1.pdf]

medRxiv preprint doi: https://doi.org/10.1101/2025.02.03.25321613; this version posted February 5, 2025. The copyright holder for this preprint (which was not certified by peer review) is the author/funder, who has granted medRxiv a license to display the preprint in perpetuity.
It is made available under a CC-BY 4.0 International license .

Supplementary Information: A Binary Prototype for Time-Series Surveillance and Intervention

Jason Olejarz, Till Hoffmann, Alex Zapf, Douaa Mugahid, Ross Molinaro, Chadwick Brown, Artem Boltyenkov, Taras Dudykevych, Ankit Gupta, Marc Lipsitch, Rifat Atun, Jukka-Pekka Onnela, Sarah Fortune, Rangarajan Sampath, Yonatan H. Grad

This Supplementary Information is organized as follows. In Section 1, we derive the optimal intervention strategy given that there is no surveillance. In Section 2, we derive the optimal surveillance and intervention strategy using the most recent bit of data. We also give an example, where we apply our framework to optimize surveillance for vector-borne pathogens. In Section 3, we derive the optimal surveillance and intervention strategy using the two most recent bits of data. We also expand on the example of the previous section. In Section 4, we explore the optimization of surveillance and intervention using the three most recent bits of data. We further demonstrate some of the intricacies that arise when optimizing surveillance using three or more bits of data.

1 No surveillance

If there is no surveillance, then the optimal strategy is either to never intervene or to always intervene. If we never intervene, then we incur an expected cost per unit time equal to a_1c. If we always intervene, then we incur a cost per unit time equal to k. Let Y represent the intervention strategy, so that $Y = 0$ if we never intervene and $Y = 1$ if we always intervene. The unnormalized expected cost per unit time, $L_0'(k;Y)$, can be expressed as

$$L_0'(k;Y) = a_1(1-Y)c + (a_1+a_0)Yk. \tag{S1}$$

Letting $K = k/c$ and $L_0(K;Y) = L_0'(k;Y)/c$ in Equation (S1), we obtain the normalized expected cost per unit time:

$$L_0(K;Y) = a_1(1-Y) + (a_1+a_0)YK. \tag{S2}$$

Using $a_1 + a_0 = 1$ in Equation (S2) and simplifying, we obtain

$$L_0(K;Y) = a_1 + (K-a_1)Y. \tag{S3}$$

Equation (S3) serves as a baseline for gauging the effectiveness of more complex surveillance and intervention strategies.

medRxiv preprint doi: https://doi.org/10.1101/2025.02.03.25321613; this version posted February 5, 2025. The copyright holder for this preprint (which was not certified by peer review) is the author/funder, who has granted medRxiv a license to display the preprint in perpetuity.
It is made available under a CC-BY 4.0 International license .

2 Surveillance using one bit of data

The unnormalized expected cost per unit time given that we use the single most recent bit of data to inform our intervention strategy, $L_1'(s_1, k; Y_0, Y_1)$, can be expressed as

$$L_1'(s_1, k; Y_0, Y_1) = s_1 + \sum_{i\in\{0,1\}} [(a_1 p_{1i})(1 - Y_i)c + (a_1 p_{1i} + a_0 p_{0i})Y_i k]. \tag{S4}$$

The terms in the summation in Equation (S4) have a simple interpretation. If we do not intervene at time t and the system is in the abnormal state at time t, then we incur a delayed cost, c. Considering the most recent bit of data, there are two ways that this can happen:

- The system can be in the abnormal state at time t, be measured as bit i at time t, and not be acted on at time t. This sequence of events occurs with probability $a_1 p_{1i}(1-Y_i)$, and i can be either 0 or 1.

If we intervene at time t, then we incur an immediate cost, k. Considering the most recent bit of data, there are four ways that this can happen:

- The system can be in the abnormal state at time t, be measured as bit i at time t, and be acted on at time t. This sequence of events occurs with probability $a_1 p_{1i} Y_i$, and i can be either 0 or 1.

- The system can be in the normal state at time t, be measured as bit i at time t, and be acted on at time t. This sequence of events occurs with probability $a_0 p_{0i} Y_i$, and i can be either 0 or 1.

Letting $K = k/c$, $S_1 = s_1/c$, and $L_1(S_1, K; Y_0, Y_1) = L_1'(s_1, k; Y_0, Y_1)/c$ in Equation (S4), we obtain the normalized expected cost per unit time:

$$L_1(S_1, K; Y_0, Y_1) = S_1 + \sum_{i\in\{0,1\}} [(a_1 p_{1i})(1 - Y_i) + (a_1 p_{1i} + a_0 p_{0i})Y_i K]. \tag{S5}$$

As shorthand notation, we define

$$\Delta_i = (1 - K)(a_1 p_{1i}) - K(a_0 p_{0i}). \tag{S6}$$

Rearranging Equation (S5), and using Equation (S6), we obtain

$$L_1(S_1, K; Y_0, Y_1) = a_1 + S_1 - \Delta_0 Y_0 - \Delta_1 Y_1. \tag{S7}$$

The task at hand is to use Equation (S7) and the observed bit sequence to guide our intervention strategy.

If $p_{01} = 0$ and $p_{11} = 1$, then we have exact knowledge of the state of the system, and the ideal intervention strategy is to intervene if and only if we observe a 1. But such a scenario is an idealization. Realistically, we expect that $p_{01} > 0$ and $p_{11} < 1$—i.e., there are nonzero probabilities of both Type I and Type II errors (Figure S1). Whenever a Type I error occurs, we unnecessarily incur an intervention cost, k. Whenever a Type II error occurs, the delayed cost of inaction, c, exceeds the immediate cost that we would have incurred had we

medRxiv preprint doi: https://doi.org/10.1101/2025.02.03.25321613; this version posted February 5, 2025. The copyright holder for this preprint (which was not certified by peer review) is the author/funder, who has granted medRxiv a license to display the preprint in perpetuity.
It is made available under a CC-BY 4.0 International license .

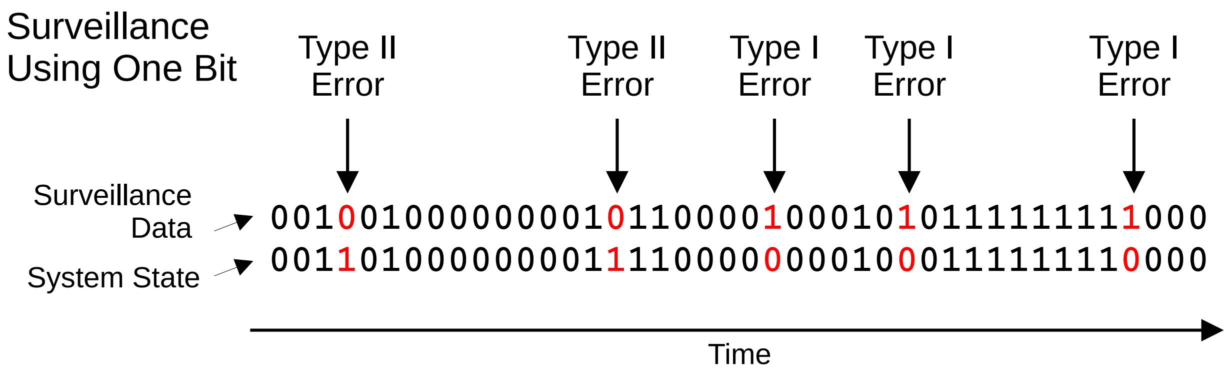

Figure S1: **Measurement errors.** Suppose that our intervention strategy is based on using only the most recent bit of data. A Type I error occurs whenever the system state is 0 and our diagnostics machine reports 1. A Type II error occurs whenever the system state is 1 and our diagnostics machine reports 0.

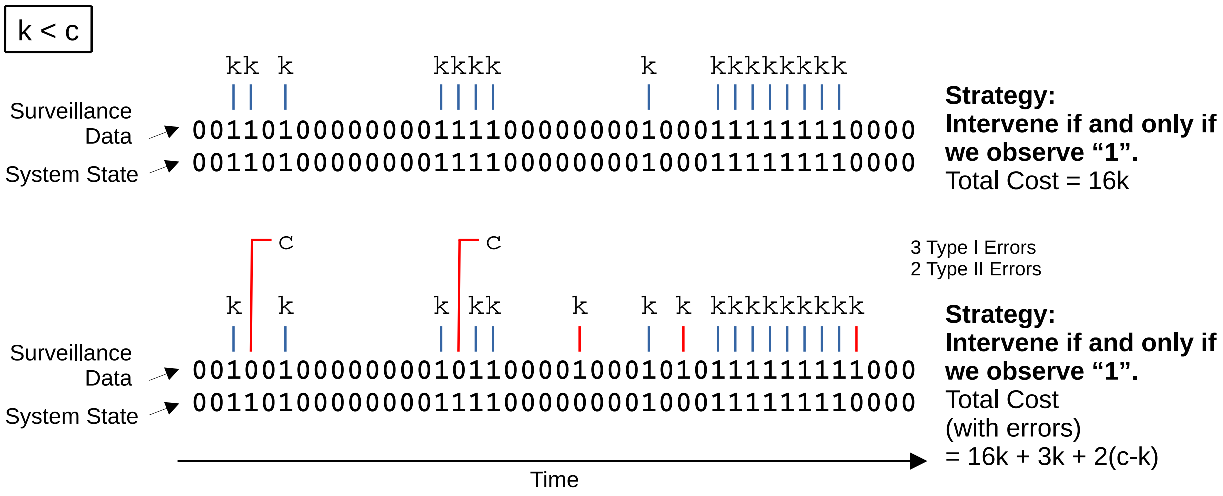

Figure S2: **Accounting for measurement errors in the calculation of total cost.** Whenever a Type I error occurs, we unnecessarily intervene and incur a cost, k. Whenever a Type II error occurs, we incur a cost, c, at a later time instead of having incurred a smaller, immediate cost k had we intervened.

medRxiv preprint doi: https://doi.org/10.1101/2025.02.03.25321613; this version posted February 5, 2025. The copyright holder for this preprint (which was not certified by peer review) is the author/funder, who has granted medRxiv a license to display the preprint in perpetuity.
It is made available under a CC-BY 4.0 International license .

intervened, k. We therefore want to minimize Type I and Type II errors (Figure S2). How should we decide what to do in response to a noisy temporal signal?

It is helpful to understand the ideal surveillance and intervention strategy depending on the values of K and S_1 (Figure 3). If K is larger than a certain value, which we denote K_+, then we should never intervene. K_+ is given by solving $L_1(0, K_+; 0, 1) = L_0(K_+; 0)$. We find

$$K_+ = \left(1 + \frac{q_{10}p_{01}}{q_{01}p_{11}}\right)^{-1}. \tag{S8}$$

If $K > K_+$, then the expected cost from reacting to false positives exceeds any benefit from averting the threat. The conclusion is that surveillance should not be executed, and intervention should never occur. K_+ is shown as the right dotted vertical line in Figure 3.

Similarly, if K is smaller than a certain value, which we denote K_-, then we should always intervene. K_- is given by solving $L_1(0, K_-; 0, 1) = L_0(K_-; 1)$. We find

$$K_- = \left(1 + \frac{q_{10}p_{00}}{q_{01}p_{10}}\right)^{-1}. \tag{S9}$$

If $K < K_-$, then the expected cost from dismissing false negatives exceeds any benefit from not intervening when there is no threat. The conclusion is that surveillance should not be executed, and intervention should always occur. K_- is shown as the left dotted vertical line in Figure 3.

K_+ and K_-, given by Equations (S8) and (S9), respectively, are therefore key quantities for determining if surveillance is beneficial. If $K_- < K < K_+$, then surveillance might be justified if surveillance costs are sufficiently low. If we use surveillance, then we incur an expected cost per unit time equal to $L_1(S_1, K; 0, 1)$. First, consider that $K > a_1$. If we don't use surveillance, then the optimal strategy is to never intervene, and we incur an expected cost per unit time equal to $L_0(K; 0)$. We solve for the value of $S_1 = S_+$ for which $L_0(K; 0) = L_1(S_+, K; 0, 1)$:

$$S_+ = \Delta_1. \tag{S10}$$

If $S_1 < S_+$, then the benefit of appropriately intervening outweighs the cost of surveillance, and surveillance is beneficial. If $S_1 > S_+$, then surveillance is too expensive and is detrimental. From Equation (S10), S_+ is plotted versus K as the upper right boundary of the green triangular region in Figure 3.

Next, consider that $K < a_1$. If we don't use surveillance, then the optimal strategy is to always intervene, and we incur an expected cost per unit time equal to $L_0(K; 1)$. We solve for the value of $S_1 = S_-$ for which $L_0(K; 1) = L_1(S_-, K; 0, 1)$:

$$S_- = K - a_1 + \Delta_1. \tag{S11}$$

If $S_1 < S_-$, then the benefit of appropriately intervening outweighs the cost of surveillance, and surveillance is beneficial. If $S_1 > S_-$, then surveillance is too expensive and is detrimental. From Equation (S11), S_- is plotted versus K as the upper left boundary of the green triangular region in Figure 3.

medRxiv preprint doi: https://doi.org/10.1101/2025.02.03.25321613; this version posted February 5, 2025. The copyright holder for this preprint (which was not certified by peer review) is the author/funder, who has granted medRxiv a license to display the preprint in perpetuity.
It is made available under a CC-BY 4.0 International license .

	$K = 0.1$	$K = 0.3$	$K = 0.4$	$K = 0.5$	$K = 0.8$
$Y = 0$	$L_0 = 0.40$	$L_0 = 0.40$	$L_0 = 0.40$	$\mathbf{L_0 = 0.40}$	$\mathbf{L_0 = 0.40}$
$Y = 1$	$\mathbf{L_0 = 0.10}$	$\mathbf{L_0 = 0.30}$	$L_0 = 0.40$	$L_0 = 0.50$	$L_0 = 0.80$
$Y_0, Y_1 = 0, 0$	$L_1 = 0.50$	$L_1 = 0.50$	$L_1 = 0.50$	$L_1 = 0.50$	$L_1 = 0.50$
$Y_0, Y_1 = 1, 0$	$L_1 = 0.44$	$L_1 = 0.56$	$L_1 = 0.62$	$L_1 = 0.68$	$L_1 = 0.86$
$Y_0, Y_1 = 0, 1$	$L_1 = 0.26$	$L_1 = 0.34$	$\mathbf{L_1 = 0.38}$	$L_1 = 0.42$	$L_1 = 0.54$
$Y_0, Y_1 = 1, 1$	$L_1 = 0.20$	$L_1 = 0.40$	$L_1 = 0.50$	$L_1 = 0.60$	$L_1 = 0.90$

Table 1: **Example of optimizing surveillance and intervention using the most recent bit of data.** We set $a_1 = 0.4$, $p_{01} = 0.2$, $p_{11} = 0.7$, and $S_1 = 0.1$.

2.1 Example using one bit of data

A simple example helps for putting the model and its potential applications into context. Many vector-borne pathogens, such as West Nile virus (WNV) and eastern equine encephalitis virus (EEEV), persist in the environment and are characterized by sudden, intermittent outbreaks. The timing of these outbreaks is extremely difficult to predict. To model this, suppose that q_{01}—defined for this example as the probability of an outbreak beginning in any given week—is small ($q_{01} \ll 1$). It is also realistic to suppose that outbreaks do not suddenly and spontaneously go away ($q_{10} \ll 1$).

Suppose that the vector population is sampled and tested weekly, and we measure the abundance of viral RNA, v, in each vector sample. When there is no outbreak, the abundance of viral RNA in each measurement is drawn from a probability distribution, $F_0(v)$. For each measurement during an ongoing outbreak, the abundance of viral RNA is drawn from a different probability distribution, $F_1(v)$. We define a cutoff value for the measured abundance of viral RNA in a sample, v'. The distributions $F_0(v)$ and $F_1(v)$ are such that the probability of observing $v \geq v'$ when there is no outbreak, p_{01}, is less than the probability of observing $v \geq v'$ when there is an ongoing outbreak, p_{11}.

If $v < v'$, then we interpret this as meaning that there is no outbreak, while if $v \geq v'$, then we suspect an ongoing outbreak and take appropriate countermeasures. The countermeasures could take many forms. For example, standing water can be drained from containers to reduce the population of mosquito larvae, and large-scale spraying of pesticides can be carried out. These actions directly target the vector population, however, which means that they would necessarily raise the value of q_{10} when applied. Since we assume q_{10} to be constant in the present treatment, we do not consider these types of interventions for this example. Rather, we consider interventions that lessen the probability of an individual contracting the pathogen, such as advising people to stay indoors and to use insect repellent when they must go outside.

These countermeasures have associated costs and benefits. By remaining indoors due to a public health advisory, people are foregoing activities that they would have otherwise engaged in. The use of insect repellent requires a non-negligible amount of money and time. But by implementing these countermeasures, we reduce the number of human infections due to the outbreak, thereby lessening pathogen-related costs due to morbidity and mortality.

The final step, then, is to understand how the aforementioned costs and benefits manifest in the model parameters k and c. In this example, k measures the costs of the intervention

medRxiv preprint doi: https://doi.org/10.1101/2025.02.03.25321613; this version posted February 5, 2025. The copyright holder for this preprint (which was not certified by peer review) is the author/funder, who has granted medRxiv a license to display the preprint in perpetuity.
It is made available under a CC-BY 4.0 International license .

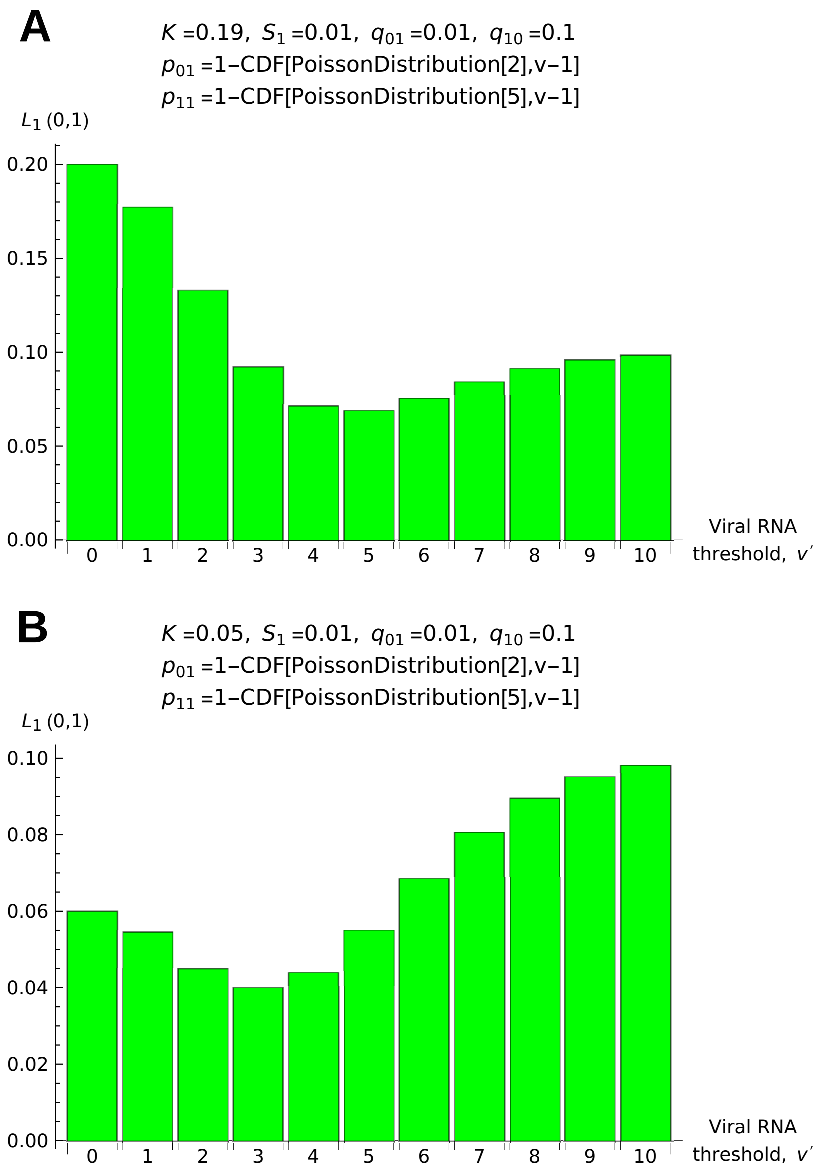

Figure S3: **Choosing the optimal viral RNA threshold.** When trapping and testing mosquitoes for arboviruses, consider that we are using only the number of infected mosquitoes in the most recent sample to inform whether we should intervene. How many infected mosquitoes, v', in the most recent sample should be required to trigger an alarm? This is determined by choosing the value of $v' = v^*$ that minimizes the loss function, $L_1(0,1)$. Intervention costs are higher in (**A**), where $v^* = 5$, than in (**B**), where $v^* = 3$. In (**A**), we should intervene if and only if there are five or more infected mosquitoes in the most recent sample, while in (**B**), we should intervene if and only if there are three or more infected mosquitoes in the most recent sample. (K: normalized intervention cost; S_1: normalized surveillance cost per unit time using one bit of data; q_{01}: probability of switching from normal to abnormal; q_{10}: probability of switching from abnormal to normal; p_{01}: probability of observing 1 while normal; p_{11}: probability of observing 1 while abnormal.)

medRxiv preprint doi: https://doi.org/10.1101/2025.02.03.25321613; this version posted February 5, 2025. The copyright holder for this preprint (which was not certified by peer review) is the author/funder, who has granted medRxiv a license to display the preprint in perpetuity.
It is made available under a CC-BY 4.0 International license .

plus the remaining costs of the pathogen despite the intervention. By contrast, c measures the costs of the pathogen given that no intervention is applied. If we are able to reconcile all types of costs using a common cost unit, then $K = k/c$ is a dimensionless number, and Equations 5 tell us if we should intervene, given a particular viral RNA threshold, v'. For pathogens such as WNV and EEEV, surveillance is routinely performed, and interventions are helpful for mitigating morbidity and mortality. Therefore, v' must be chosen to be not too low and not too high, so that Equations 5 give us $Y_0 = 0$ and $Y_1 = 1$. But what value of v' minimizes $L_1(S_1, K; Y_0, Y_1)$? This value of v', which we denote v^*, is the optimal viral RNA threshold for triggering intervention. Figure S3 demonstrates the calculation of v^*.

3 Surveillance using two bits of data

The unnormalized expected cost per unit time given that we use the two most recent bits of data, $L_2'(s_2, k; Y_{00}, Y_{10}, Y_{01}, Y_{11})$, can be expressed as

$$\begin{aligned} L_2'(s_2, k; Y_{00}, Y_{10}, Y_{01}, Y_{11}) &= s_2 \\ &+ \sum_{i,j\in\{0,1\}} [(a_1 p_{1i} q_{11} p_{1j} + a_0 p_{0i} q_{01} p_{1j})(1 - Y_{ij})c \\ &+ (a_1 p_{1i} q_{11} p_{1j} + a_0 p_{0i} q_{01} p_{1j} + a_0 p_{0i} q_{00} p_{0j} + a_1 p_{1i} q_{10} p_{0j}) Y_{ij} k]. \end{aligned} \tag{S12}$$

The terms in the summation in Equation (S12) have a simple interpretation. If we do not intervene at time t and the system is in the abnormal state at time t, then we incur a delayed cost, c. Considering the two most recent bits of data, there are eight ways that this can happen:

- The system can be in the abnormal state at time $t-1$, be measured as bit i at time $t-1$, remain in the abnormal state at time t, be measured as bit j at time t, and not be acted on at time t. This sequence of events occurs with probability $a_1 p_{1i} q_{11} p_{1j}(1-Y_{ij})$, and i and j can each be either 0 or 1.

- The system can be in the normal state at time $t-1$, be measured as bit i at time $t-1$, transition to the abnormal state at time t, be measured as bit j at time t, and not be acted on at time t. This sequence of events occurs with probability $a_0 p_{0i} q_{01} p_{1j}(1-Y_{ij})$, and i and j can each be either 0 or 1.

If we intervene at time t, then we incur an immediate cost, k. Considering the two most recent bits of data, there are sixteen ways that this can happen:

- The system can be in the abnormal state at time $t-1$, be measured as bit i at time $t-1$, remain in the abnormal state at time t, be measured as bit j at time t, and be acted on at time t. This sequence of events occurs with probability $a_1 p_{1i} q_{11} p_{1j} Y_{ij}$, and i and j can each be either 0 or 1.

- The system can be in the normal state at time $t-1$, be measured as bit i at time $t-1$, transition to the abnormal state at time t, be measured as bit j at time t, and be acted on at time t. This sequence of events occurs with probability $a_0 p_{0i} q_{01} p_{1j} Y_{ij}$, and i and j can each be either 0 or 1.

medRxiv preprint doi: https://doi.org/10.1101/2025.02.03.25321613; this version posted February 5, 2025. The copyright holder for this preprint (which was not certified by peer review) is the author/funder, who has granted medRxiv a license to display the preprint in perpetuity.
It is made available under a CC-BY 4.0 International license .

- The system can be in the normal state at time $t-1$, be measured as bit i at time $t-1$, remain in the normal state at time t, be measured as bit j at time t, and be acted on at time t. This sequence of events occurs with probability $a_0 p_{0i} q_{00} p_{0j} Y_{ij}$, and i and j can each be either 0 or 1.

- The system can be in the abnormal state at time $t-1$, be measured as bit i at time $t-1$, transition to the normal state at time t, be measured as bit j at time t, and be acted on at time t. This sequence of events occurs with probability $a_1 p_{1i} q_{10} p_{0j} Y_{ij}$, and i and j can each be either 0 or 1.

Letting $S_2 = s_2/c$ and $L_2(S_2, K; Y_{00}, Y_{10}, Y_{01}, Y_{11}) = L_2'(s_2, k; Y_{00}, Y_{10}, Y_{01}, Y_{11})/c$ in Equation (S12), we obtain the normalized expected cost per unit time:

$$\begin{aligned} &L_2(S_2, K; Y_{00}, Y_{10}, Y_{01}, Y_{11}) = S_2 \\ &+ \sum_{i,j\in\{0,1\}} [(a_1 p_{1i} q_{11} p_{1j} + a_0 p_{0i} q_{01} p_{1j})(1 - Y_{ij}) \\ &+ (a_1 p_{1i} q_{11} p_{1j} + a_0 p_{0i} q_{01} p_{1j} + a_0 p_{0i} q_{00} p_{0j} + a_1 p_{1i} q_{10} p_{0j}) Y_{ij} K]. \end{aligned} \tag{S13}$$

As shorthand notation, we define

$$\Delta_{ij} = (1-K)(a_1 p_{1i} q_{11} p_{1j} + a_0 p_{0i} q_{01} p_{1j}) - K(a_1 p_{1i} q_{10} p_{0j} + a_0 p_{0i} q_{00} p_{0j}). \tag{S14}$$

Rearranging Equation (S13), and using Equation (S14), we obtain

$$L_2(S_2, K; Y_{00}, Y_{10}, Y_{01}, Y_{11}) = a_1 + S_2 - \Delta_{00} Y_{00} - \Delta_{10} Y_{10} - \Delta_{01} Y_{01} - \Delta_{11} Y_{11}. \tag{S15}$$

The task at hand is to use Equation (S15) and the observed bit sequence to guide our intervention strategy.

For some applications, surveillance using the two most recent bits of data can be detrimental. In Figure S4, for a particular sequence of system states and observed bits, we compare the strategy to intervene if and only if we observe 1 to the strategy to intervene if and only if we observe 11. For the latter strategy, the number of Type I errors is reduced but the number of Type II errors is substantially increased, such that requiring an observation of 11 in succession for deciding to intervene is counterproductive. In Figure S5, for the same sequence of system states and observed bits, we compare the strategy to intervene if and only if we observe 1 to the strategy to not intervene if and only if we observe 00. For the latter strategy, the number of Type II errors is reduced but the number of Type I errors is substantially increased, such that requiring an observation of 00 in succession for deciding to not intervene is counterproductive.

Another example shows how surveillance using the two most recent bits of data can be optimal (Table 2). If intervention costs are low, then it might be beneficial to not intervene if and only if both of the most recent bits are 0. By observing 00 in succession, we are more confident that the system is in the normal state and that intervention is useless. If intervention costs are high, then it might be beneficial to intervene if and only if both of the most recent bits are 1. By observing 11 in succession, we are more confident that the system is in the abnormal state and that intervention is worthwhile.

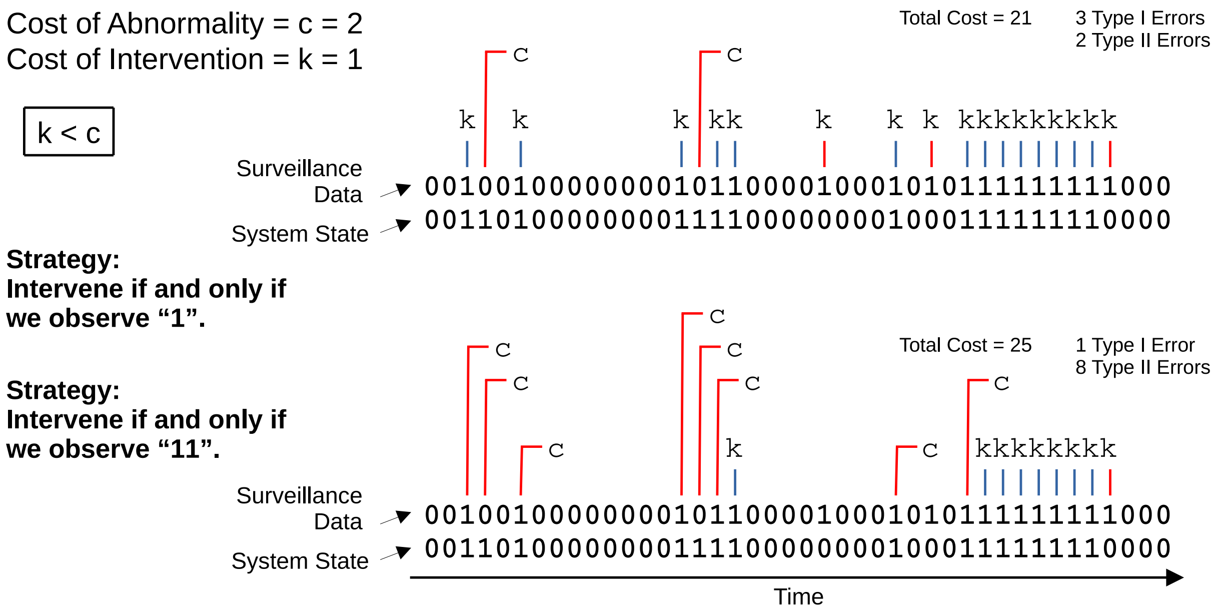

Figure S4: **Requiring reinforcement for intervening.** For this particular sequence of system state values and measurements, we compare the strategy to intervene if and only if we observe 1 versus the strategy to intervene if and only if we observe 11 in succession. For the latter strategy, there are two fewer Type I errors but six more Type II errors. The additional Type II errors mean that the latter strategy would have performed worse than the former for this realization of the dynamics.

medRxiv preprint doi: https://doi.org/10.1101/2025.02.03.25321613; this version posted February 5, 2025. The copyright holder for this preprint (which was not certified by peer review) is the author/funder, who has granted medRxiv a license to display the preprint in perpetuity.
It is made available under a CC-BY 4.0 International license .

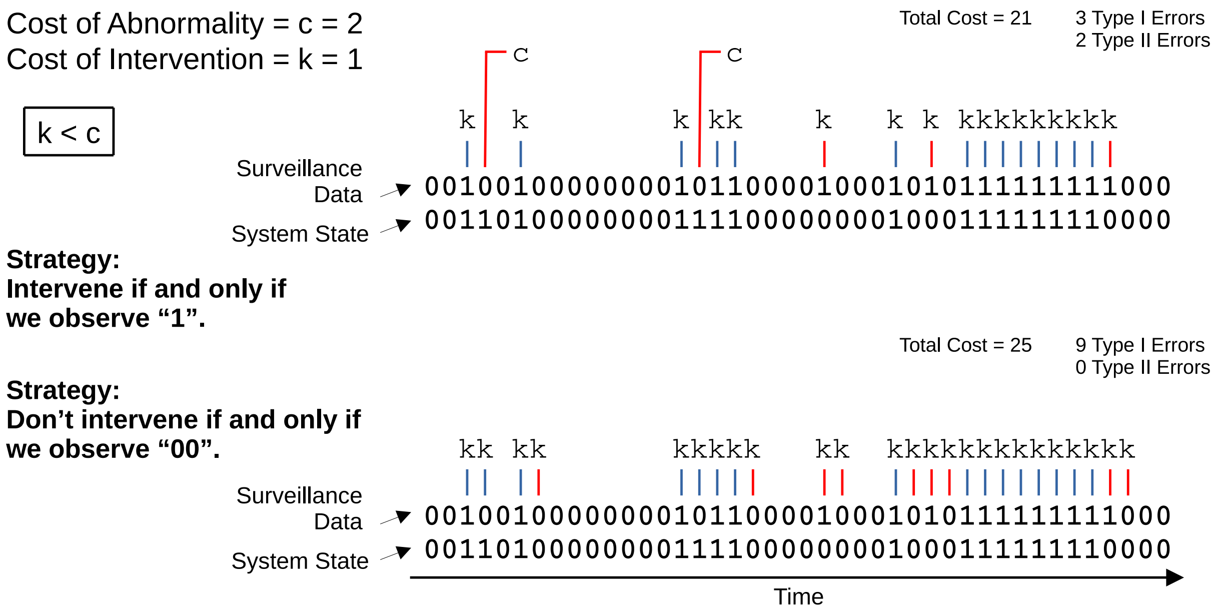

Figure S5: **Requiring reinforcement for not intervening.** For this particular sequence of system state values and measurements, we compare the strategy to intervene if and only if we observe 1 versus the strategy to not intervene if and only if we observe 00 in succession. For the latter strategy, there are two fewer Type II errors but six more Type I errors. The additional Type I errors mean that the latter strategy would have performed worse than the former for this realization of the dynamics.

medRxiv preprint doi: https://doi.org/10.1101/2025.02.03.25321613; this version posted February 5, 2025. The copyright holder for this preprint (which was not certified by peer review) is the author/funder, who has granted medRxiv a license to display the preprint in perpetuity.
It is made available under a CC-BY 4.0 International license .

	$K = 0.3$	$K = 0.6$
$Y = 0$	$L_0 = 0.40000$	$L_0 = 0.40000$
$Y = 1$	$L_0 = 0.30000$	$L_0 = 0.60000$
$Y_0, Y_1 = 0, 0$	$L_1 = 0.40100$	$L_1 = 0.40100$
$Y_0, Y_1 = 1, 0$	$L_1 = 0.40500$	$L_1 = 0.60900$
$Y_0, Y_1 = 0, 1$	$L_1 = 0.29700$	$L_1 = 0.39300$
$Y_0, Y_1 = 1, 1$	$L_1 = 0.30100$	$L_1 = 0.60100$
$Y_{00}, Y_{10}, Y_{01}, Y_{11} = 0, 0, 0, 0$	$L_2 = 0.40200$	$L_2 = 0.40200$
$Y_{00}, Y_{10}, Y_{01}, Y_{11} = 1, 0, 0, 0$	$L_2 = 0.42596$	$L_2 = 0.56792$
$Y_{00}, Y_{10}, Y_{01}, Y_{11} = 0, 1, 0, 0$	$L_2 = 0.38204$	$L_2 = 0.44408$
$Y_{00}, Y_{10}, Y_{01}, Y_{11} = 1, 1, 0, 0$	$L_2 = 0.40600$	$L_2 = 0.61000$
$Y_{00}, Y_{10}, Y_{01}, Y_{11} = 0, 0, 1, 0$	$L_2 = 0.34604$	$L_2 = 0.40808$
$Y_{00}, Y_{10}, Y_{01}, Y_{11} = 1, 0, 1, 0$	$L_2 = 0.37000$	$L_2 = 0.57400$
$Y_{00}, Y_{10}, Y_{01}, Y_{11} = 0, 1, 1, 0$	$L_2 = 0.32608$	$L_2 = 0.45016$
$Y_{00}, Y_{10}, Y_{01}, Y_{11} = 1, 1, 1, 0$	$L_2 = 0.35004$	$L_2 = 0.61608$
$Y_{00}, Y_{10}, Y_{01}, Y_{11} = 0, 0, 0, 1$	$L_2 = 0.35396$	$\mathbf{L_2 = 0.38792}$
$Y_{00}, Y_{10}, Y_{01}, Y_{11} = 1, 0, 0, 1$	$L_2 = 0.37792$	$L_2 = 0.55384$
$Y_{00}, Y_{10}, Y_{01}, Y_{11} = 0, 1, 0, 1$	$L_2 = 0.33400$	$L_2 = 0.43000$
$Y_{00}, Y_{10}, Y_{01}, Y_{11} = 1, 1, 0, 1$	$L_2 = 0.35796$	$L_2 = 0.59592$
$Y_{00}, Y_{10}, Y_{01}, Y_{11} = 0, 0, 1, 1$	$L_2 = 0.29800$	$L_2 = 0.39400$
$Y_{00}, Y_{10}, Y_{01}, Y_{11} = 1, 0, 1, 1$	$L_2 = 0.32196$	$L_2 = 0.55992$
$Y_{00}, Y_{10}, Y_{01}, Y_{11} = 0, 1, 1, 1$	$\mathbf{L_2 = 0.27804}$	$L_2 = 0.43608$
$Y_{00}, Y_{10}, Y_{01}, Y_{11} = 1, 1, 1, 1$	$L_2 = 0.30200$	$L_2 = 0.60200$

Table 2: **Example of optimizing surveillance and intervention using the two most recent bits of data.** We set $q_{01} = 0.2$, $q_{10} = 0.3$, $p_{01} = 0.2$, $p_{11} = 0.5$, $S_1 = 0.001$, and $S_2 = 0.002$.

medRxiv preprint doi: https://doi.org/10.1101/2025.02.03.25321613; this version posted February 5, 2025. The copyright holder for this preprint (which was not certified by peer review) is the author/funder, who has granted medRxiv a license to display the preprint in perpetuity.
It is made available under a CC-BY 4.0 International license .

We have already worked out the conditions for when surveillance using one bit is superior to no surveillance. When we include the possibility of using two bits of data to guide our intervention, determination of the optimal surveillance and intervention strategy becomes more intricate. We must still consider how surveillance using one bit performs versus no surveillance, as shown in Figure 3 and Figure 4A. We must similarly determine if surveillance using two bits is superior to no surveillance, and we must determine if surveillance using two bits is superior to surveillance using one bit.

When comparing surveillance using two bits to no surveillance, there are multiple comparisons that must be done separately. First, we compare the strategy to intervene if and only if we observe 11 to never intervening. If K is larger than a certain value, which we denote K_{0+}, then we should never intervene. K_{0+} is given by solving $L_2(0, K_{0+}; 0, 0, 0, 1) = L_0(K_{0+}; 0)$. We find

$$K_{0+} = \left[1 + \left(\frac{1}{K_+} - 1\right)\left(\frac{q_{00}p_{01} + q_{01}p_{11}}{q_{11}p_{11} + q_{10}p_{01}}\right)\right]^{-1}. \tag{S16}$$

If $K > K_{0+}$, then the expected cost from reacting to false positives whenever we observe 11 exceeds any benefit from averting the threat.

Similarly, we compare the strategy to intervene if and only if we observe 11 to always intervening. If K is smaller than a certain value, which we denote K_{0-}, then we should always intervene. K_{0-} is given by solving $L_2(0, K_{0-}; 0, 0, 0, 1) = L_0(K_{0-}; 1)$. We find

$$K_{0-} = \left[1 + \left(\frac{1}{K_-} - 1\right)\left(\frac{p_{00}p_{10} + p_{10}p_{01}(q_{00}p_{00} + q_{01}p_{10})}{p_{00}p_{10} + p_{00}p_{11}(q_{11}p_{10} + q_{10}p_{00})}\right)\right]^{-1}. \tag{S17}$$

If $K < K_{0-}$, then the expected cost from dismissing false negatives whenever we do not observe 11 exceeds any benefit from not intervening when there is no threat.

If $K_{0-} < K < K_{0+}$, then using two bits and intervening if and only if we observe 11 might be justified if surveillance costs are sufficiently low. If we use surveillance, then we incur a cost per unit time equal to $L_2(S_2, K; 0, 0, 0, 1)$. First, consider that $K > a_1$. If we don't use surveillance, then the optimal strategy is to never intervene, and we incur a cost per unit time equal to $L_0(K; 0)$. We set $L_0(K; 0) = L_2(S_{0+}, K; 0, 0, 0, 1)$ and solve for S_{0+}:

$$S_{0+} = \Delta_{11}. \tag{S18}$$

If $S_2 < S_{0+}$, then the benefit of appropriately intervening whenever we observe 11 outweighs the cost of surveillance, and surveillance using two bits is beneficial relative to never intervening. If $S_2 > S_{0+}$, then surveillance using two bits is too expensive relative to never intervening. From Equation (S18), S_{0+} is plotted versus K as the upper right boundary of the triangular region in Figure 4B.

Next, consider that $K < a_1$. If we don't use surveillance, then the optimal strategy is to always intervene, and we incur a cost per unit time equal to $L_0(K; 1)$. We set $L_0(K; 1) = L_2(S_{0-}, K; 0, 0, 0, 1)$ and solve for S_{0-}:

$$S_{0-} = K - a_1 + \Delta_{11}. \tag{S19}$$

If $S_2 < S_{0-}$, then the benefit of appropriately intervening whenever we observe 11 outweighs the cost of surveillance, and surveillance using two bits is beneficial relative to always intervening. If $S_2 > S_{0-}$, then surveillance using two bits is too expensive relative to always

medRxiv preprint doi: https://doi.org/10.1101/2025.02.03.25321613; this version posted February 5, 2025. The copyright holder for this preprint (which was not certified by peer review) is the author/funder, who has granted medRxiv a license to display the preprint in perpetuity.
It is made available under a CC-BY 4.0 International license .

intervening. From Equation (S19), S_{0-} is plotted versus K as the upper left boundary of the triangular region in Figure 4B.

Next, we compare the strategy to not intervene if and only if we observe 00 to never intervening. If K is larger than a certain value, which we denote K_{1+}, then we should never intervene. K_{1+} is given by solving $L_2(0, K_{1+}; 0, 1, 1, 1) = L_0(K_{1+}; 0)$. We find

$$K_{1+} = \left[1 + \left(\frac{1}{K_+} - 1\right)\left(\frac{p_{11}p_{01} + p_{00}p_{11}(q_{00}p_{01} + q_{01}p_{11})}{p_{11}p_{01} + p_{10}p_{01}(q_{11}p_{11} + q_{10}p_{01})}\right)\right]^{-1}. \tag{S20}$$

If $K > K_{1+}$, then the expected cost from reacting to false positives whenever we do not observe 00 exceeds any benefit from averting the threat.

Similarly, we compare the strategy to not intervene if and only if we observe 00 to always intervening. If K is smaller than a certain value, which we denote K_{1-}, then we should always intervene. K_{1-} is given by solving $L_2(0, K_{1-}; 0, 1, 1, 1) = L_0(K_{1-}; 1)$. We find

$$K_{1-} = \left[1 + \left(\frac{1}{K_-} - 1\right)\left(\frac{q_{00}p_{00} + q_{01}p_{10}}{q_{11}p_{10} + q_{10}p_{00}}\right)\right]^{-1}. \tag{S21}$$

If $K < K_{1-}$, then the expected cost from dismissing false negatives whenever we observe 00 exceeds any benefit from not intervening when there is no threat.

If $K_{1-} < K < K_{1+}$, then using two bits and not intervening if and only if we observe 00 might be justified if surveillance costs are sufficiently low. If we use surveillance, then we incur a cost per unit time equal to $L_2(S_2, K; 0, 1, 1, 1)$. First, consider that $K > a_1$. If we don't use surveillance, then the optimal strategy is to never intervene, and we incur a cost per unit time equal to $L_0(K; 0)$. We set $L_0(K; 0) = L_2(S_{1+}, K; 0, 1, 1, 1)$ and solve for S_{1+}:

$$S_{1+} = \Delta_{10} + \Delta_{01} + \Delta_{11}. \tag{S22}$$

If $S_2 < S_{1+}$, then the benefit of appropriately intervening whenever we do not observe 00 outweighs the cost of surveillance, and surveillance using two bits is beneficial relative to always intervening. If $S_2 > S_{1+}$, then surveillance using two bits is too expensive relative to always intervening. From Equation (S22), S_{1+} is plotted versus K as the upper right boundary of the triangular region in Figure 4C.

Next, consider that $K < a_1$. If we don't use surveillance, then the optimal strategy is to always intervene, and we incur a cost per unit time equal to $L_0(K; 1)$. We set $L_0(K; 1) = L_2(S_{1-}, K; 0, 1, 1, 1)$ and solve for S_{1-}:

$$S_{1-} = K - a_1 + \Delta_{10} + \Delta_{01} + \Delta_{11}. \tag{S23}$$

If $S_2 < S_{1-}$, then the benefit of appropriately intervening whenever we do not observe 00 outweighs the cost of surveillance, and surveillance using two bits is beneficial relative to always intervening. If $S_2 > S_{1-}$, then surveillance using two bits is too expensive relative to always intervening. From Equation (S23), S_{1-} is plotted versus K as the upper left boundary of the triangular region in Figure 4C.

We must also determine if surveillance using two bits is superior to surveillance using one bit. If K is between two values, which we denote K_{2+} and K_{2-}, then we

medRxiv preprint doi: https://doi.org/10.1101/2025.02.03.25321613; this version posted February 5, 2025. The copyright holder for this preprint (which was not certified by peer review) is the author/funder, who has granted medRxiv a license to display the preprint in perpetuity.
It is made available under a CC-BY 4.0 International license .

should intervene if and only if we observe 1. K_{2+} is equal to the value of K for which $L_2(S_1, K_{2+}; 0, 0, 0, 1) = L_1(S_1, K_{2+}; 0, 1)$. We find

$$K_{2+} = \left[1 + \left(\frac{1}{K_+} - 1\right)\left(\frac{q_{00}p_{00} + q_{01}p_{10}}{q_{11}p_{10} + q_{10}p_{00}}\right)\right]^{-1}. \tag{S24}$$

K_{2-} is equal to the value of K for which $L_2(S_1, K_{2-}; 0, 1, 1, 1) = L_1(S_1, K_{2-}; 0, 1)$. We find

$$K_{2-} = \left[1 + \left(\frac{1}{K_-} - 1\right)\left(\frac{q_{00}p_{01} + q_{01}p_{11}}{q_{11}p_{11} + q_{10}p_{01}}\right)\right]^{-1}. \tag{S25}$$

For $K > K_{2+}$, surveillance using two bits is superior to surveillance using one bit if $S_2 - S_1 < S_{2+}$, where S_{2+} is given by $L_2(S_{2+}, K; 0, 0, 0, 1) = L_1(0, K; 0, 1)$. We have

$$S_{2+} = \Delta_{11} - \Delta_1. \tag{S26}$$

For $K < K_{2-}$, surveillance using two bits is superior to surveillance using one bit if $S_2 - S_1 < S_{2-}$, where S_{2-} is given by $L_2(S_{2-}, K; 0, 1, 1, 1) = L_1(0, K; 0, 1)$. We have

$$S_{2-} = \Delta_{10} + \Delta_{01} + \Delta_{11} - \Delta_1. \tag{S27}$$

From Equations (S26) and (S27), S_{2+} and S_{2-} are plotted versus K as the right and left boundary lines, respectively, in Figure 4D.

The understanding of surveillance using two bits is summarized concisely in the four panels of Figure 4. What can we learn from this plot? For simplicity in the descriptions that follow, suppose that all surveillance costs are negligible.

One might first ask: If we must decide between no surveillance and surveillance using two bits, where we intervene if and only if we observe 11, then what should we do? There are two K values, K_{0+} and K_{0-}, that answer this question. For context, it is insightful to compare these quantities to K_+ and K_-, respectively. From inspecting Equation (S16), K_{0+} is necessarily larger than K_+. This is because when we require observing 11 to intervene, we are reducing the rate of false positives, so for interventions for which $K_+ < K < K_{0+}$, false positives are easier to tolerate, and it makes sense to have surveillance. From inspecting Equation (S17), K_{0-} is necessarily larger than K_-. This is because when we require observing 11 to intervene, we are increasing the rate of false negatives, so for interventions for which $K_- < K < K_{0-}$, it makes sense to always intervene, thereby averting any costs due to missed detection.

One might then ask: If we must decide between no surveillance and surveillance using two bits, where we do not intervene if and only if we observe 00, then what should we do? There are two K values, K_{1+} and K_{1-}, that answer this question. From inspecting Equation (S20), K_{1+} is necessarily smaller than K_+. This is because when we require observing 00 to not intervene, we are increasing the rate of false positives, so for interventions for which $K_{1+} < K < K_+$, it makes sense to never intervene, thereby averting any costs due to unwarranted intervention. From inspecting Equation (S21), K_{1-} is necessarily smaller than K_-. This is because when we require observing 00 to not intervene, we are reducing the rate of false negatives, so for interventions for which $K_{1-} < K < K_-$, false negatives are easier to tolerate, and it makes sense to have surveillance.

medRxiv preprint doi: https://doi.org/10.1101/2025.02.03.25321613; this version posted February 5, 2025. The copyright holder for this preprint (which was not certified by peer review) is the author/funder, who has granted medRxiv a license to display the preprint in perpetuity.
It is made available under a CC-BY 4.0 International license .

One might also ask: If we must decide between surveillance using one bit and surveillance using two bits, then what should we do? There are two K values, K_{2+} and K_{2-}, that answer this question. From inspecting Equations (S24) and (S25), K_{2+} is necessarily smaller than K_+, and K_{2-} is necessarily larger than K_-. Therefore, if $K_+ < K < 1$, then we should never intervene, and if $0 < K < K_-$, then we should always intervene. Further, if $K_{2+} < K < K_+$, then intervention is sufficiently costly that surveillance using two bits—with the strategy to intervene if and only if we observe 11—is helpful for reducing false positives and unnecessary intervention costs. Also, if $K_- < K < K_{2-}$, then intervention is sufficiently cheap that surveillance using two bits—with the strategy to not intervene if and only if we observe 00—is helpful for reducing false negatives and costs due to missed detection. But if $K_{2-} < K < K_{2+}$, then surveillance using two bits is inferior to surveillance using one bit. For these values of K, when considering the strategy to intervene if and only if we observe 11, the benefit of averting false positives is outweighed by the cost of false negatives. Also for these values of K, when considering the strategy to not intervene if and only if we observe 00, the benefit of averting false negatives is outweighed by the cost of false positives.

3.1 Example using two bits of data

Using two measurements in succession, how should we decide whether or not to intervene? It is worth noting that a proper decision rule in this setting would use all available information, which would include the viral RNA abundance measured in the samples from both the previous week and the current week. But for applying this model, we use only a single bit of data to represent each weekly measurement. If $v \geq v'$ for the previous week, then the previous week's observation is 1, and 0 otherwise. If $v \geq v'$ for the current week, then the current week's observation is 1, and 0 otherwise. Given a particular viral RNA threshold, v', Equations 8 determine the ideal intervention strategy, and we must choose the optimal value of v', given by v^*, such that $L_2(S_2, K; Y_{00}, Y_{10}, Y_{01}, Y_{11})$ is minimized. A simple demonstration considering strategies that use either one or two bits of data, again in the context of surveillance for arboviruses, is shown in Figure S6.

4 Example using three bits of data

When considering surveillance using more than two bits of data, calculation of the optimal strategy is more complicated. For example, consider two possible bit sequences: 001 and 110. In the former, the most recent measurement is 1, while the two previous measurements are both 0. In the latter, the most recent measurement is 0, while the two previous measurements are both 1. For determining the intervention strategy, does the most recent observation of 1 or 0 outweigh the two previous observations of 00 or 11, or vice versa?

Figure S7 shows two possibilities for different parameter sets. First, consider Figure S7A for the case of large intervention costs (inside the gray region). A 1 in the most recent measurement outweighs 0 readings in both of the two previous measurements, and we should intervene if we observe 001. Also, a 0 in the most recent measurement outweighs two 1 readings in both of the two previous measurements, and we should not intervene if we observe 110. Thus, the analysis simplifies: Since a measurement of 001 warrants intervention, mea-

medRxiv preprint doi: https://doi.org/10.1101/2025.02.03.25321613; this version posted February 5, 2025. The copyright holder for this preprint (which was not certified by peer review) is the author/funder, who has granted medRxiv a license to display the preprint in perpetuity.
It is made available under a CC-BY 4.0 International license .

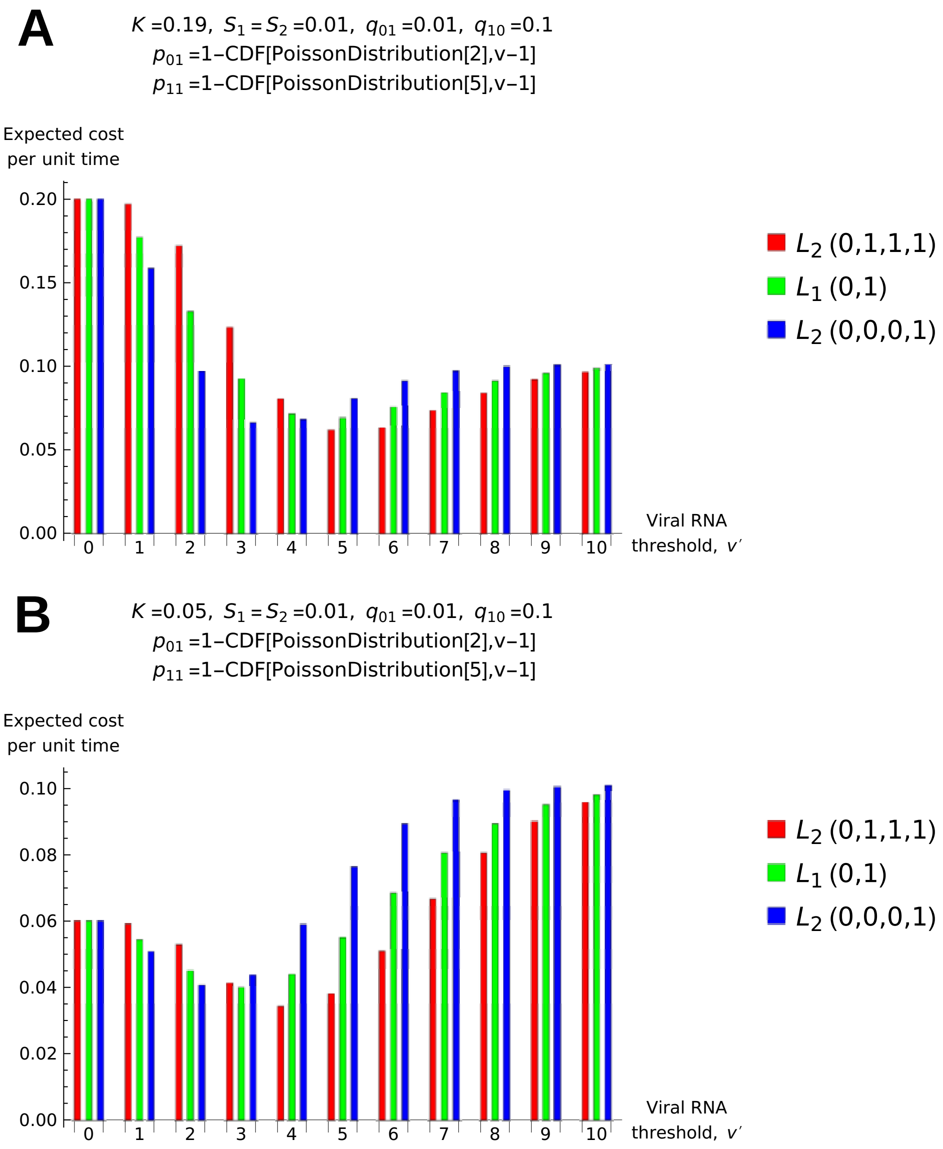

Figure S6: **Choosing the optimal viral RNA threshold and the optimal number of bits to use.** The parameter values are the same as in Figure S3. However, here, we consider two additional strategies: intervene if and only if the two most recent bits are 1, and intervene if and only if at least one of the two most recent bits is 1. The corresponding loss functions are $L_2(0,0,0,1)$ and $L_2(0,1,1,1)$, respectively. In both (**A**) and (**B**), the strategy to use only a single bit of data is outperformed by the strategy to intervene if and only if at least one of the two most recent bits is 1. In (**A**), we have $v^* = 5$, and we should intervene if and only if at least one of the two most recent samples contained five or more mosquitoes. In (**B**), we have $v^* = 4$, and we should intervene if and only if at least one of the two most recent samples contained four or more mosquitoes. (K: normalized intervention cost; $S_1 = S_2$: normalized surveillance cost per unit time using one or two bits of data; q_{01}: probability of switching from normal to abnormal; q_{10}: probability of switching from abnormal to normal; p_{01}: probability of observing 1 while normal; p_{11}: probability of observing 1 while abnormal.)

medRxiv preprint doi: https://doi.org/10.1101/2025.02.03.25321613; this version posted February 5, 2025. The copyright holder for this preprint (which was not certified by peer review) is the author/funder, who has granted medRxiv a license to display the preprint in perpetuity.
It is made available under a CC-BY 4.0 International license .

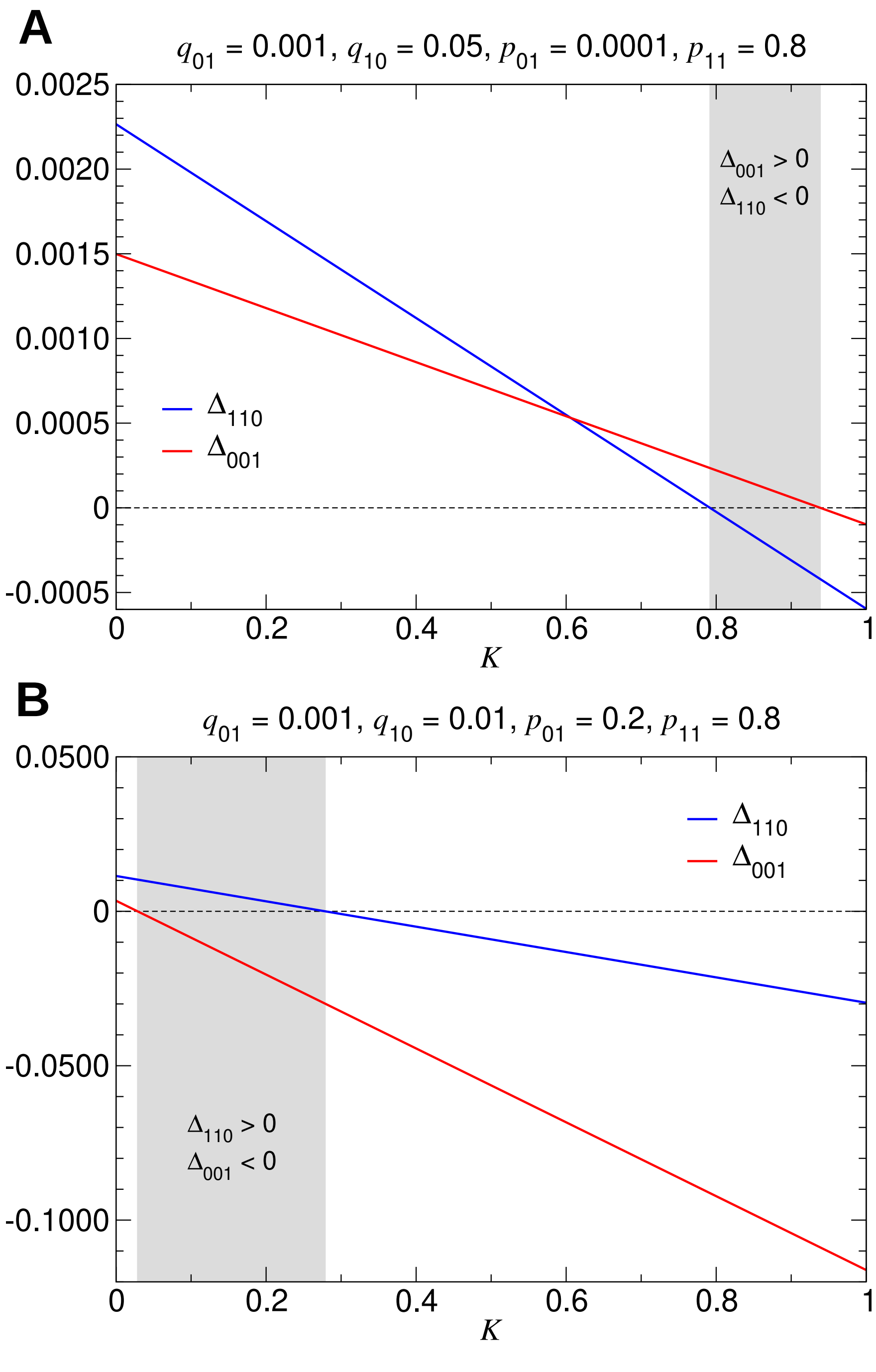

Figure S7: **Surveillance using three bits of data.** In (**A**), for intervention costs within the gray region, we should intervene if we observe 001 and not intervene if we observe 110. In (**B**), for intervention costs within the gray region, we should intervene if we observe 110 and not intervene if we observe 001. This example illustrates that optimization of surveillance and intervention using more than two bits of data can be intricate. (q_{01}: probability of switching from normal to abnormal; q_{10}: probability of switching from abnormal to normal; p_{01}: probability of observing 1 while normal; p_{11}: probability of observing 1 while abnormal.)

medRxiv preprint doi: https://doi.org/10.1101/2025.02.03.25321613; this version posted February 5, 2025. The copyright holder for this preprint (which was not certified by peer review) is the author/funder, who has granted medRxiv a license to display the preprint in perpetuity.
It is made available under a CC-BY 4.0 International license .

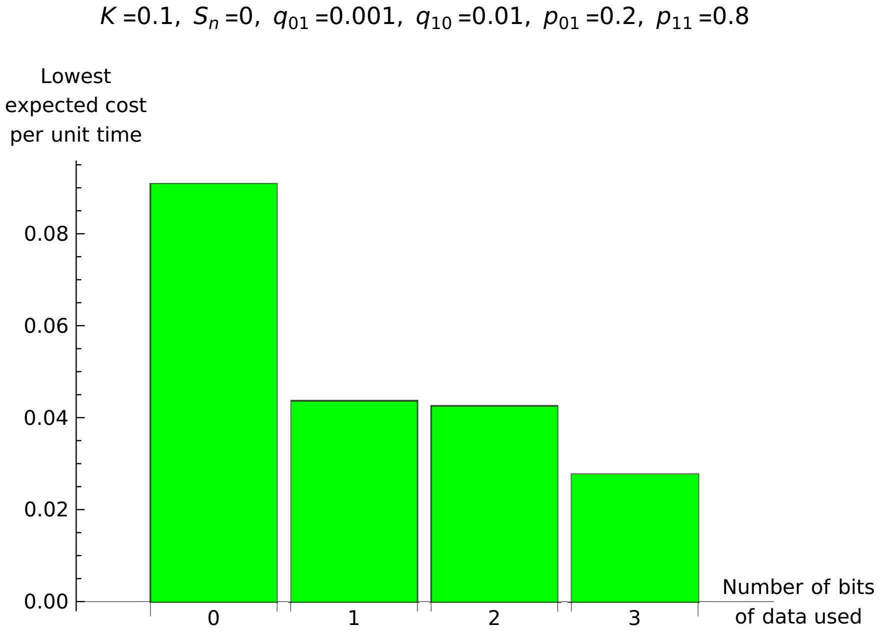

Figure S8: **Determining the optimal amount of data to use.** Consider the lowest expected cost per unit time that is achievable by using zero, one, two, or three bits of data in the decision to intervene. Using one bit of data yields a large improvement over no surveillance, using two bits of data yields a marginal improvement over using one bit of data, and using three bits of data yields a large improvement over using two bits of data. (K: normalized intervention cost; S_n: normalized surveillance cost per unit time using n bits of data; q_{01}: probability of switching from normal to abnormal; q_{10}: probability of switching from abnormal to normal; p_{01}: probability of observing 1 while normal; p_{11}: probability of observing 1 while abnormal.)

surements of 011, 101, or 111 also warrant intervention. Furthermore, since a measurement of 110 should not induce an intervention, measurements of 100, 010, or 000 also should not induce an intervention. The conclusion is that the optimal strategy is to use only the most recent bit of data, and we should intervene if and only if this bit is 1.

By contrast, consider Figure S7B for small intervention costs (inside the gray region). Two previous 1 readings in succession outweigh a 0 in the most recent measurement, and we should intervene if we observe 110. Also, two previous 0 readings in succession outweigh a 1 in the most recent measurement, and we should not intervene if we observe 001. Using the three most recent bits of data, the optimal strategy is that we should intervene if and only if we observe 110, 101, 011, or 111. This example shows that care must be taken when determining the optimal strategy if more than two bits of data are being used.

A further consideration is the number of bits of data to use for deciding whether to intervene. For the same parameter values as in Figure S7B, Figure S8 shows the lowest cost per unit time that is achievable when using zero, one, two, or three bits of data. Using zero bits of data corresponds to no surveillance and no intervention, and the resulting expected cost per unit time provides a benchmark. By using just a single bit of data, and by intervening if and only if we observe 1, we achieve a $>50\%$ reduction in the expected cost per unit time.

medRxiv preprint doi: https://doi.org/10.1101/2025.02.03.25321613; this version posted February 5, 2025. The copyright holder for this preprint (which was not certified by peer review) is the author/funder, who has granted medRxiv a license to display the preprint in perpetuity.
It is made available under a CC-BY 4.0 International license .

Using two bits of data and intervening if and only if we observe 11 delivers only a marginal benefit over using one bit of data. However, using three bits of data delivers a dramatic improvement in performance over using two bits of data. Determination of the optimal number of bits of data to use is therefore not always an intuitive exercise, and care must be taken in doing so.
